# The Biochemistry of Phytocannabinoids and Metabolic Engineering of Their Production in Heterologous Systems

**DOI:** 10.3390/ijms22052454

**Published:** 2021-02-28

**Authors:** Kaitlyn Blatt-Janmaat, Yang Qu

**Affiliations:** 1 Department of Chemistry, University of New Brunswick, Fredericton, NB E3B 5A3, Canada; kaitlyn.blatt.janmaat@unb.ca; 2 Department of Chemical Engineering, University of New Brunswick, Fredericton, NB E3B 5A3, Canada

**Keywords:** cannabinoid biosynthesis, metabolic engineering, synthetic biology, yeast fermentation, polyketide synthase, aromatic prenyltransferase, FAD-dependent monooxygenase

## Abstract

The medicinal properties of cannabis and the its legal status in several countries and jurisdictions has spurred the massive growth of the cannabis economy around the globe. The value of cannabis stems from its euphoric activity offered by the unique phytocannabinoid tetrahydrocannabinol (THC). However, this is rapidly expanding beyond THC owing to other non-psychoactive phytocannabinoids with new bioactivities that will contribute to their development into clinically useful drugs. The discovery of the biosynthesis of major phytocannabinoids has allowed the exploration of their heterologous production by synthetic biology, which may lead to the industrial production of rare phytocannabinoids or novel synthetic cannabinoid pharmaceuticals that are not easily offered by cannabis plants. This review summarizes the biosynthesis of major phytocannabinoids in detail, the most recent development of their metabolic engineering in various systems, and the engineering approaches and strategies used to increase the yield.

## 1. Introduction

**Figure 1:**
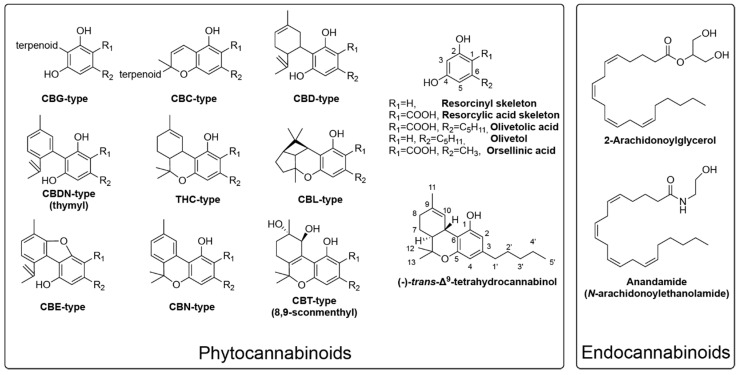
Representative structures for endocannabinoids, phytocannabinoids, intermediates and numbering system.

Phytocannabinoids are meroterpenoids (i.e., partial terpenoid derivative) with a resorcinyl core featuring a *para* position isoprenyl, alkyl, or aralkyl side chain produced as specialized metabolites in plants (Figure 1) [1,2]. The most well-studied phytocannabinoid producer, and namesake of the chemical family, is *Cannabis sativa. C. sativa* has been historically cultivated and utilized for thousands of years for food, textiles, and its medicinal properties. When humans transitioned to a sedentary lifestyle due to the rise of agriculture, intensive cultivation and breeding of *C. sativa* for fibers began [3]. Over time, the psychoactive property of the crop was discovered and selection of desirable euphoric activity began [4]. Due to extensive breeding, a variety of *C. sativa* cultivars exist that can be distinguished by their morphology and chemical profiles [5]. Hemp, or fiber type cannabis, has a low Δ⁹-tetrahydrocannabinol (THC) content and grows quite tall with minimal branching while marijuana, or drug type cannabis, is shorter, has a higher degree of branching, and a high THC content.

To date, over 100 phytocannabinoids have been identified in *C. sativa* [1,6]. These compounds are separated into various classes based on their structures: cannabigerol (CBG)-type, cannabichromene (CBC)-type, cannabidiol (CBD)-type, thymyl-type, THC-type, cannabicyclol (CBL)-type, cannabielsoin (CBE)-type, cannabinol (CBN)-type, and 8,9-sconmenthyl-type [7], among which only the THC type has been known to possess the psychoactive property (Figure 1). In *C. sativa*, all phytocannabinoids have an alkyl side chain attached to the central resorcinyl core. Phytocannabinoids are primarily synthesized and stored within glandular trichomes that are present on the female flowers and to a lower degree on the leaves [8]. These trichomes contain resin storage cells where cannabinoids and terpenes build up to form a resin that is postulated to act as an antiherbivory agent. As the flower and seeds mature, the composition of cannabinoids and terpenes within the resin changes, with the highest phytocannabinoid content found at flower maturity [8]. Phytocannabinoids are not transported to seeds, with only minute quantities (< 5ppm) found in hemp seed oil [9].

THC may be unique to cannabis, however phytocannabinoids based on prenylated resorcinols have been found in other plant and microbial species, which is nicely summarized in several recent reviews [10,11]. Specifically, perrottetinene is a bibenzyl phytocannabinoid from liverwort *Radula* spp. which is structurally similar to THC except for the *cis*-stereochemistry within the heteroaromatic ring system and the addition of an aromatic side-chain [12,13]. In addition, the non-cyclized, CBG type phytocannabinoids and cyclized, CBC-type phytocannabinoids are also reported in *Rhododendron* spp. (Ericaceae family)*, Helichrysum umbraculigerum* (Asteraceae family)*,* and some fungal species [10,11,14,15]. The sporadic discoveries of these compounds perhaps suggest a convergent evolution contributed by metabolic plasticity from the common polyketide formation/aromatic prenylation. These biosynthetic steps are described in detail in later sections.

The psychotropic effect of THC led to the discovery of the human cannabinoid type 1 (CB1) receptor, followed by the discovery of the CB2 receptor based on sequence homology. Both receptors are membrane-bound G-protein coupled receptor (GPCR), and the studies on them led to the discovery of the endocannabinoid ligands such as anandamide and 2-arachidonoylglycerol (2-AG) responsible for their activation and modulation (Figure 1). The details can be found in several excellent reviews [16,17,18]. CB1, the main target of THC-type phytocannabinoids, is found in the central and peripheral nervous system, the liver, the reproductive system, the cardiovascular system, the skeletal muscles, and in the GI tract. The diversity of the locations of the receptor suggests the diversity of the functions it may serve in these locations. The psychotropic effects of CB1 are mainly produced by its retrograde inhibition on both excitatory and inhibitory terminals of presynaptic neurons. Once activated, CB1 suppresses the release of neurotransmitters by inhibiting voltage-gated Ca^2+^ channels to reduce ion influx and by inhibiting adenylyl cyclase to stop the signaling pathway. CB2, the less understood of the receptors, is located mainly on immune-modulating cells where it modulates the release of cytokine and impacts immune cell migration. In addition to the cannabinoid receptors, phytocannabinoids are found to interact or modulate the responses of several other receptors such as the transient receptor potential cation channel subfamily V member 1 (TPRV1), the serotonin receptors, and a few other GPCRs [16,17,18,19]. Alteration of CB1 and CB2 levels have been found to be associated with neurodegenerative diseases such as Alzheimer’s, Parkinson’s, and Huntington’s disease, which could provide a novel avenue of treatment. Mitochondrial CB1 was also found to regulate mitochondrial metabolism in the brain, which suggests it may serve an additional role in memory formation [20,21]. Additionally, targeting the endocannabinoid system may also be useful for psychiatric diseases such as schizophrenia and neuromuscular disorders [16,17,18].

While targeting the endocannabinoid system shows great promise for novel treatments, THC’s CB1 agonism is considered a major limit to its clinical use due to the psychoactive property. On the other hand, the increasing evidence of CBD’s neuromodulatory role, especially in epilepsy, suggests that further studies on other non-psychoactive phytocannabionids may lead to their development into clinically useful drugs [16,17,18]. The potential for a class of phytocannabinoid-based pharmaceuticals, the legal status of recreational cannabis in several jurisdictions, and the anticipation of full cannabis legalization at federal level in the United States and the European Union, have together accelerated the basic and applied research on cannabis and phytocannabinoids. Producing cannabinoids without cannabis cultivation may provide an alternative route to access pharmaceutically relevant structures that are scarce or not produced in plants. This review summarizes the biochemistry of phytocannabinoids in *C. sativa* and the most recent development of phytocannabinoid production engineering in heterologous systems, from both peer-reviewed articles and publicly available patent and patent applications because of the significant commercial interest.

## 2. Critical Biosynthetic Steps for Phytocannabinoid Formation

In cannabis, three critical steps are required to divert the flux from the primary metabolism to form individual cannabinoids, which include the formation of olivetolic acid through polyketide biosynthesis and cyclization, olivetolic acid prenylation to form cannabigerolic acid (CBGA), and cyclization reactions to form three major cannabinoid structures. Because of enzyme patent status and commercial importance, each step is discussed in details with respect to biochemistry and enzyme engineering in the below three sections (Section 2.1, Section 2.2 and Section 2.3).

### 2.1. The Formation of Olivetolic Acid

**Figure 2:**
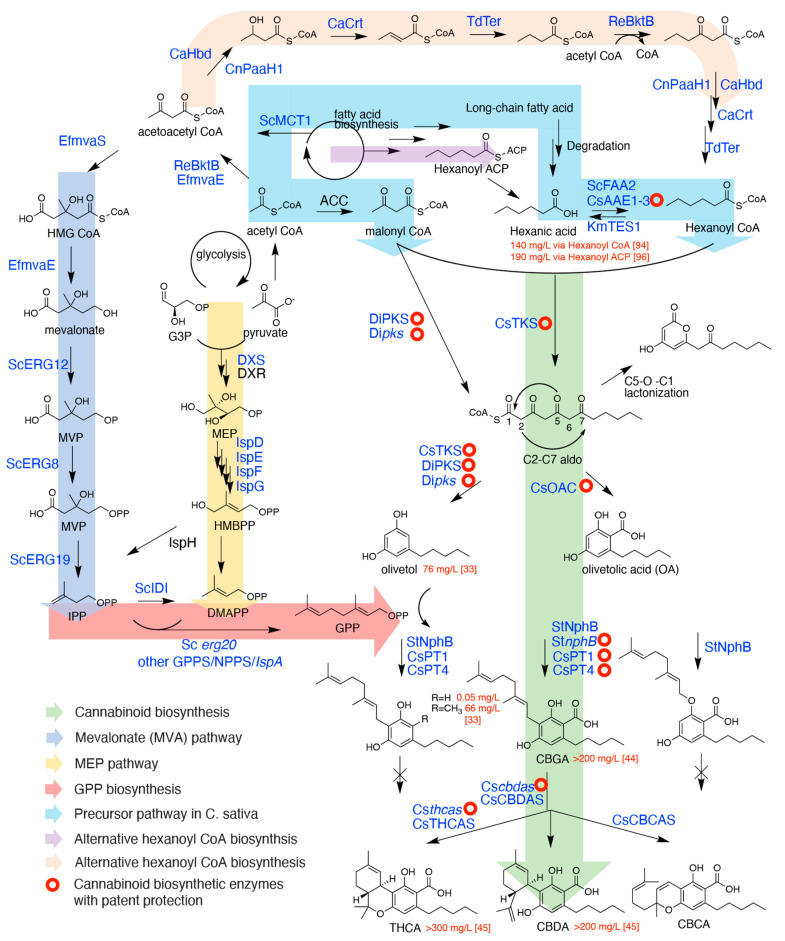
The native and engineered biosynthetic pathway for phytocannabionids in *C. sativa*. Enzymes in italics are mutant enzymes. The abbreviations are listed as follows. The cannabinoid pathway: TKS, tetraketide synthase; OAC, olivetolic acid synthase; PKS, polyketide synthase; NphB, naphterpin biosynthetic cluster gene B; PT, prenyltransferase; THCAS, tetrahydrocannabinolic acid synthase; CBDAS, cannabidiolic acid synthase; CBCAS, cannabichromenic acid synthase. MVA pathway: mvaE, acetoacetyl-CoA thiolase/HMG-CoA reductase; mvaS, HMG-CoA synthase; ERG12, mevalonate kinase; ERG8, phosphomevalonate kinase; ERG19, diphosphomevalonate decarboxylase; IDI1 isopentenyl diphosphate:dimethylallyl diphosphate isomerase; ERG20, farnesyl pyrophosphate synthetase. MEP pathway: DXS, deoxyxylulose 5-phosphate synthase; DXR, deoxyxylulose 5-phosphate reductoisomerase; IspD, 4-diphosphocytidyl-2-C-methylerythritol (CDP-ME) synthase; IspE, CDP-ME kinase; IspF, 2-*C*-methyl-*D*-erythritol 2,4-cyclodiphosphate synthase; IspG, (E)-4-Hydroxy-3-methyl-but-2-enyl pyrophosphate (HMB-PP) synthase; IspH, HMB-PP reductase. Fatty acid biosynthesis: BktB, β-keto thiolase; ACC, acetyl CoA carboxylase; MCT1: malonyl CoA-acyl carrier protein transacylase; HBD/PaaH1, β-hydroxybutyryl-CoA de-hydrogenase; CRT: 3-hydroxybutyryl-CoA dehydratase; Ter, trans-enoyl-CoA reductase; FAA2, Long-chain-fatty-acid--CoA ligase 2; KmTES1, *Kluyveromyces marxianus* acyl-CoA thioesterase; AAE: acyl activating enzyme. Species abbreviations: Cs, *Cannabis sativa*; St, *Streptomyces* strain CL109; Di, *Dictyostelium discoideum*; Ef, *Enterococcus faecalis*; Re, *Ralstonia eutropha*; Ca, *Clostridium acetobutylicum*; Td, *Treponema denticola*; Sc, *Saccharomyces cerevisiae*; Km, *Kluyveromyces marxianus*.

The first committed step in cannabinoid biosynthesis in *C. sativa* is formation of a polyketide intermediate and its cyclization to form olivetolic acid (OA) (Figure 2). In *C. sativa*, this reaction is catalyzed by a type III polyketide synthase named tetraketide synthase (CsTKS) or olivetol synthase (CsOLS), and a unique cyclase named olivetolic acid cyclase (CsOAC), which are both enriched in the glandular trichomes [22,23,24,25]. In the reaction of CsTKS, malonyl coenzyme A (CoA) is first decarboxylated and then the resulting acetyl forms a C-C bond with the carbonyl of the enzyme-bound hexanoyl CoA. After three rounds of condensation, the labile tetraketide intermediate is released, which is cyclized by OAC via C2-C7 aldol condensation (–H_2_O) followed by spontaneous aromatization to form the resorcylic acid skeleton (Figure 1) while still retaining the CoA thioester. Spontaneous ester hydrolysis occurs to give the final product OA (Figure 2).

Plant polyketide biosynthesis is largely catalyzed by type III PKSs that condense several malonyl CoA with various starting CoA esters followed by a cyclization. Type III PKSs are considered the minimal PKS as they are simple homodimers with only polyketide synthase and cyclase activities. In contrast, type I PKSs are large polypeptides with several domains such as acyl carrier proteins, acyltransferases, PKSs, dehydrogenases, and keto-reductases that catalyze a series of reactions. Type II PKSs are instead individual enzymes that forms a complex functionally similar to Type I PKSs. More detailed biochemistry of PKS can be found in these excellent reviews [26,27,28].

The most ubiquitous type III PKS in plants is perhaps chalcone synthase (CHS), the first committed enzyme in the biosynthesis of the flavonoid metabolite class, which is found in all plants. After forming the labile polyketide, CHS cyclizes it to form the chalcone skeleton via C1-C6 Claisen condensation (Figure 2), which is one of the two most common cyclization mechanisms among type III PKSs. In contrast, stilbene synthase cyclizes the polyketide chain via the C2-C7 aldol condensation with concomitant elimination of the terminal carbonyl as CO_2_. In this reaction, the tetraketide thioester intermediate must be hydrolyzed to the carboxylate prior to the aldol condensation while still being docked in the PKS active site [29,30]. In fact, early release of hydrolyzed polyketide intermediate can also cause spontaneous lactonization via C1-O5 bond formation seen in the formation of many triacetic lactone (TAL) polyketides (Figure 2). While TAL products have not been reported in cannabis, they are found in other plant species such as *Gerbera hybrida* (ornamental daisy) [27]. Without CsOAC, CsTKS proceeds with the regular aldol condensation mechanism to form olivetol (resorcinol skeleton) instead of OA (resorcylic acid skeleton). This carboxylate group is presented in the final cannabinoids such as Δ⁹-tetrahydrocannabinolic acid (THCA), which renders it non-psychoactive. When heated, THCA loses this carboxylate and transforms to the psychoactive THC. As such, it would appear that forming olivetol instead of OA is beneficial for producing the psychoactive THC instead to THCA. However, the presence of this carboxylate group is necessary for the enzyme activity of the terminal cylcases that forms various cannabinoids [31]. Therefore, OA formation via cyclization by CsOAC is required for cyclic cannabinoid biosynthesis.

In rare cases, PKS can catalyze the C2-C7 aldol condensation prior to thiol esterification, which retains the terminal carbonyl without an additional cyclase. To date, there is only a handful such PKSs that are cloned and characterized, including the 2′-oxoalkylresorcylic acid synthase (ORAS) from the fugus *Neurospora crassa* [29], alkylresorcylic acid synthase 1 and 2 (ARAS1, 2) from rice [30], and the stilbene carboxylic acid synthase (STCS) from *Hydrangea macrophylla* [32]. While these enzymes produce their respective resorcylic acids as the major products, resorcinols were also produced, at least *in vitro*. It has also been reported that the PKS orcinol synthase from *Rhododendron dauricum* produced small amounts of orsellinic acid (Figure 1). Orsellinic acid is an OA analog with a methyl side-chain instead of the pentyl group found in OA. It may be possible to engineer CsTKS to function directly as an OA synthase without the help of CsOAC. However, such modifications have yet to be reported, and CsOAC remains required for the biosynthesis of the final cyclized cannabinoids.

A patent application describes the use of a type I fatty acid synthase/PKS from the amoeba *Dictyostelium discoideum* for the biosynthesis of olivetol, 1-methylolivetol, and their geranylated products CBG and methyl-CBG (Figure 2) [33]. The DiPKS is a large multi-domain enzyme with β-ketoacyl-synthase, acyl transacetylase, dehydratase, *C*-methyl transferase, enoyl reductase, ketoreductase, acyl carrier protein, and polyketide synthase activities, which synthesized 1-methylolivetol directly from malonyl CoA, therefore sparing the need of additional engineering or exogenous supply of hexanoyl CoA. A G1516R mutation could effectively knock out the *C*-methyltransferase activity leaving olivetol as the only product, whereas G1516D_G1518A double mutation allows a mixture of methyl and non-methyl olivetol formation. When DiPKS was expressed in their engineered yeast *Saccharomyces cerevisiae*, 76 mg/L olivetol and 66 mg/L methyl-CBG *de novo* production was reported (Table 1). The prevalence of resorcinol structures in nature also suggests that other PKSs may be recruited for olivetol production. While CBG could not be cyclized to THC, CBD or other final cannabinoids by the characterized *C. sativa* cyclases [31], it is not known whether these enzymes can accept methyl-CBG as a substrate.

Other than the implication of the DiPKS, all other publicly available peer-reviewed articles and patent applications for cannabinoid biosynthesis use CsTKS and CsOAC to our knowledge. The duo can accept a number of natural or modified aliphatic CoA starters to form the respective resorcylic acids of different side-chain lengths (e.g., C3-C6 etc.) [14,22,37]. The later pathway enzymes (prenyltransferase and cyclases) were also promiscuous enough to adopt the different side-chains, since novel cyclized cannabinoids were formed by feeding different carboxylate acid starting molecules to yeast expressing these genes [37]. These results make it possible to further diversify cannabinoids by altering the side-chain.

Unlike the membrane-bound prenyltransferase or the terminal cyclases requiring a eukaryotic protein folding system (discussed later in this article), expression of CsTKS and CsOAC is straight forward in both bacterial or eukaryotic systems. Currently, OA formation by CsTKS and CsOAC has not been actively engineered in yeast or *E. coli*, compared to the late prenylation and cyclization reactions and the early geranyl diphosphate (GPP) and hexanoyl CoA supply. However, a recent report indicated that OA strongly inhibited CsTKS activity at medium (0.25 mM) and high (5 mM) OA concentrations in their in vitro cannabinoid production system, suggesting protein engineering of CsTKS may be required to improve its catalysis [56]. When assayed in vitro, the recombinant CsTKS and CsOAC have always produced significant amounts of lactone by-products (TALs), which are not reported in *C. sativa* [22,23,24]. While the reason is unclear, it may be possible to engineer the recombinant proteins for improved product specificity.

### 2.2. Cannabigerolic Acid Formation by Aromatic Prenyltransferases

Aromatic prenyltransferases are responsible for geranylating OA to form CBGA that is further cyclized to different cannabinoids. All known plant aromatic *C*-prenyltransferases are membrane-bound enzymes that are classified as *p*-hydroxybenzoic acid (PHB) and homogentisate (HG) PTs [57]. OA prenyltransferases for CBGA biosynthesis and chrysoeriol prenyltransferases for cannflavinA/B biosynthesis all belong to the HG family that are localized to plant plastids [38,57,58]. The plastid membrane localization strongly suggests the prenyl donors are supplied by the plastid 2-*C*-methyl-*D*-erythritol 4-phosphate (MEP) pathway (Figure 2), and the hydrophobic interior of the phospholipid bilayers may also facilitate the hydrophobic product release after prenylation.

OA prenyltransferase activity in *C. sativa* was first described in the crude, soluble protein extract from hemp leaves [59]. Interestingly, the attempt of using membrane fractions from the crude protein was not successful. The crude, soluble proteins were able to prenylate OA at C3 with GPP or its *cis*-isomer neryl diphosphate (NPP), but could not prenylate olivetol [59] (Figure 1). Since then, two membrane-bound OA prenyltransferases, namely CsPT1 and CsPT4 sharing 62% sequence identity at amino acid level, have been cloned from *C. sativa* and characterized. While only accepting a GPP donor, CsPT1 could prenylate a broad array of aromatic acceptors including OA, olivetol, phlorisovalerophenone (precursor to bitter acids in hops), naringenin (flavonoid), and resveratrol (stilbenoid) [60]. When OA was used, CBGA was reported as the major product with a minor product identified as 5-genanylolivetolic acid [60]. CsPT4 could geranylate OA and a number of its C6 alkyl substituted analogs to CBGA and analogs [37], suggesting this substitution does not significantly impact substrate binding or catalysis. However, its substrate specificity towards other aromatic compounds such as olivetol or prenyl donors such as NPP has not been reported. The remaining *C. sativa* hypothetical aromatic prenyltransferases (CsPT2,3,5–10) were reported to be ineffective for the prenylation of OA [37,38], while CsPT3 was identified as the cannflavin A/B synthase that prenylates chrysoeriol [58].

CsPT4 however showed markedly superior enzyme activity *in vitro*, with a K_m_ for OA at 6.72 µM [37] comparing to 60 mM documented for CsPT1 [60], which raises the question as which PT is physiologically responsible for CBGA formation. There are enzymes with unusually high K_m_, such as the grape *N*-methylanthranilate synthase that has a K_m_ of 15 mM for its co-substrate methanol. The plant makes up the deficiency by overproduction of the enzyme and through the high methanol content that occurs during fruit maturation [61]. The expression of CsPT4 in *C. sativa* was enriched in glandular trichomes [38], whereas CsPT1 expression was also enriched in trichomes, flowers, and young leaves where cannabinoids were accumulated [38,60]. While further evidence is required to determine which PT of these two is responsible for cannabinoid biosynthesis *in planta*, it is still puzzling considering the original report of this prenylation activity was discovered using soluble proteins from leaves. Nevertheless, both PTs were able to geranylate OA in vivo in *S. cerevisiae* as demonstrated by OA feeding in several reports [34,35,36,37,51]. The activity of CsPT1 was also shown when expressed in *E. coli* [43,46], with a CBGA yield of 1.2 mg/L in growth media by feeding OA and GPP (Table 1) [43]. However, the in vivo activity of CsPT1 was reported very poor or inactive in *S. cerevisiae*, the methylotrophic yeast *Komagataella phaffii* (formerly known as *Pichia pastoris*) and *Nicotiana benthamiana* (tobacco) [37,38,41].

Feeding OA to *S. cerevisiae* expressing CsPT4 has led to 96 mg/L and 216 mg/L CBGA production in the growth media from two patent applications (Table 1) [34,35], whereas in vivo CBGA formation by CsPT1 was either reported much lower or not described with titers. A green fluorescent protein (GFP)-dPT4(1–246nt deletion) fusion protein or full-length PT4 were used in each case. The yield differences may have resulted from the *de novo* GPP supply in each background, although other factors might be involved too. The first strain included a mutant ScERG20 (F96W, N127W) as GPP synthase (GPPS), an *N*-terminal-truncated 3-hydroxy-3-methyl-glutaryl-coenzyme A synthase (tScHMG1), and the deletion of yeast native ERG20 promoter [34]. The second strain had extensive engineering in the mevalonate pathway, which included overexpression of seven *S. cerevisiae* genes (tHMG1, ERG8, 10, 12, 13, 19, isopentenyl diphosphate isomerase) and the pyruvate decarboxylase (PDC) from *Zymomonas mobilis*, in addition to ERG20(F96W, N127W) [35]. It was evident that ERG20(F96W, N127W) was optimal in yeast compared to other tested plant GPPS such as those from *Abies grandis* (grand fir) and *Phalaenopsis bellina* (orchid), which afforded as high as 15 mg/L CBGA from OA feeding [35]. Site-directed mutagenesis has not been reported for either prenyltransferases, yet it was mentioned that an upcoming patent application describes the CsPT4 *N*-terminal engineering and domain swapping with aromatic prenyltransferase from hops to improve its activity [35].

In addition to the two *C. sativa* membrane-bound aromatic prenyltransferases, a soluble aromatic prenyltransferase NphB from the bacteria *Streptomyces* strain CL109 was shown to geranylate OA to CBGA [41]. StNphB was discovered and characterized from a gene cluster related with the biosynthesis of naphterpin, a polyketide with cyclized geranyl chain similar to that of THC [62]. Although the native substrate of NphB has not been determined [63], it exhibited high degree of substrate promiscuity, catalyzing both *C*- and *O*-geranylation of a number of aromatic compounds including hydroxynaphthalenes, flavonoids, stilbenoids, and olivetol (K_m_ 0.52 mM, *k_cat_* 0.027×10^3^/s) [62,64]. When expressed in the yeasts *S. cerevisiae* and *K. phaffii*, StNphB could catalyze the OA geranylation at both C3 forming CBGA (minor) and O2 forming 2-*O*-geranylolivetolic acid (2OG, major) (Figure 2) [41]. Prenylation on other positions of the aromatic ring was also reported [65]. Co-expression of vacuole-targeted THCA synthase (THCAS) and StNphB of multiple genomic copies in *K. phaffii* enabled the in vivo biotransformation of OA and GPP to CBGA and THCA [41].

The wild-type (WT) StNphB was reported to have much lower in vivo and in vitro activity towards OA than CsPT4 when expressed in yeast [33,37,41,52] and *E. coli* (K_m_ 0.64 mM, *k_cat_* 0.0021/min) [54,55]. Several groups have worked on enhancing its product specificity and kinetics. Mutants of Y288A_G286S or Y288V_A232S dramatically improved StNphB kinetics resulting K_m_ 0.45 mM, *k_cat_* 1.58/min, and K_m_ 0.12 mM, *k_cat_* 1.30/min for OA [54,55]. Such point mutations also rendered almost exclusive production of CBGA, which together led to 21-fold increase in CBGA titer (744 mg/L) in a cell free system with OA feedstock and *de novo* produced GPP (Table 1). By continuously removing CBGA from the cell free reaction with nonane, the group was able to push the CBGA titer to 1.25g/L. Further mutations were performed to improve the mutant StNphB thermal stability [56]. Improvement of CBGA product specificity was also achieved by Q295F mutation resulting in 20-fold increase of in vivo activity in *E. coli* and concomitant CBGA/2OG ratio shift from 1:5 to 20:1 with GPP/OA feedstock [66]. A large-scale mutagenesis of StNphB also revealed altered activity and regiospecificity of the aromatic ring prenylation [65]. The WT StNphB has also been shown to geranylate 1-methylolivetol and olivetol in vivo, allowing production of 66 mg/L methyl-CBG and 0.05 mg/L CBG *de novo* under the same condition [33].

It is possible that prenyltransferases from other species may also have CBGA synthase (CBGAS) activity. It has been reported that 12 out of 35 tested aromatic PT enzymes exhibited in vitro CBGAS activity from the bacteria and fungi species of *Aspergillus terreus*, *Aspergillus fischeri*, *Actinobacteria bacterium*, and *streptomyces* spp. [67]. Whether they are suitable for cannabinoid production is yet to be reported. Currently, no other plant prenyltransferases have been reported with CBGAS activity.

### 2.3. FAD-Depended Cyclases Form Various Cannabinoids

In *C. sativa*, CBGA is cyclized by several homologous flavin adenine dinucleotide (FAD)-dependent monoxygenases, namely THCA, CBDA, and CBCA synthase (THCAS, CBDAS, and CBCAS) which share ca. 80% amino acid sequence identities [50,68,69,70,71,72]. By initiating a hydride shift to the oxidized FAD prosthetic group, these enzymes facilitate the oxidative cyclization of CBGA or its *cis*-isomer cannabinerolic acid (CBNRA) to the respective products. Molecular oxygen is reduced to hydrogen peroxide to regenerate the oxidized FAD in a reaction mechanism similar to that of the Berberine Bridge Enzyme (BBE) in the benzylisoquinoline alkaloid biosynthetic pathway [73]. These enzymes do not accept CBG as a substrate, since the interaction of carboxylate group with a histidine and an adjacent tyrosine residue is important for substrate binding as demonstrated by site directed mutagenesis and reported crystal structures [31]. Albeit forming the respective product as their name suggests, all three enzymes were shown to form small amounts of other cannabinoids when tested in vitro and *in vivo*, which was also influenced by pH [36,72,74]. The major by-product of THCAS is CBCA (ca. 10% at optimal pH), whereas the major by-products of CBDAS are THCA and CBCA (ca. 3–6% at optimal pH) [72]. The non-stringent product specificity is also likely an intrinsic feature of the native enzymes in *C. sativa*, because THC has always been detected in hemp varieties such as Finola that does not contain a THCAS in its genome [75]. In many cases, small amounts of enzyme by-products are not an issue. However, with THC being a controlled, psychoactive substance in most jurisdictions, the concomitant THCA production during CBDA production may be perceived as a problem for the cannabis industry who is looking for THC-void solutions. This intrinsic by-product production also suggests that creating a THC-void cannabis variety may not be fruitful without genetic modifications.

Similar to other BBE-like enzymes, these cyclases are known for the requirement of protein *N*-glycosylation and intramolecular disulfide bridges, which are believed to be required for proper polypeptide folding and possibly the recruitment of the covalently bound FAD. This is supported by the fact that none of the BBE-like enzymes have been expressed as an active enzyme in *E. coli*, a prokaryote with an *N*-glycosylation process fundamentally different from that of eukaryotes. Expression of the recombinant proteins in tobacco was also only possible when they were tagged for secretion or endoplasmic reticulum (ER) localization, whereas the deletion of the signal peptides or replacement of a chloroplast targeting signal peptide did not yield detectable proteins [74]. However, the *N*-glycans are not needed for enzyme activity after protein maturation, since deglycosylated THCAS still showed comparable activity in vitro [39]. The known *C. sativa* cyclases all contain a 28 amino acid, *N*-terminal signal peptide, and do not possess the classic C-terminal ER retention signal. The signal peptide directs the nascent polypeptides through the plant secretory pathway via ER translocation, *N*-glycosylation, disulfide bonds forming, FAD linking, and protein folding in ER lumen, *N*-glycan modifications in Golgi, and vesicle medicated secretion to the apoplastic region (i.e., outside of a cell) of the secretory cells of the glandular trichomes [76]. Such localization is perhaps evolutionarily preferential, as the hydrophobic environment in the glandular trichome storage cavity allows optimal enzymatic activity and accumulation of the hydrophobic products to concentrations that are cytotoxic to plant cells [76]. When expressed in tobacco leaves, the recombinant proteins were also detected in the chloroplast in addition to the apoplast [74]. Additionally, the signal peptide is cleaved in the mature enzyme. Although the recruitment process for FAD is not clear, considerable knowledge has been made with regard to the *N*-glycosylation and disulfide formation that involve numerous chaperones and enzymes, which are nicely summarized in several reviews [77,78,79,80]. While mechanistically the *N*-glycosylation process in ER lumen is highly conserved among plants, animals, and fungi, the recognition and glycan attachments may differ significantly in different systems, which impacts the expression of *C. sativa* cyclases in fungi such as yeast. Incorrect or insufficient *N*-glycosylation can result in protein sorting into ER-associated degradation (ERAD) [78], and overexpression of secretory proteins may overload the *N*-glycosylation system which results in ER stress and unfolded protein response (UPR) that limits the recombinant protein expression [81].

Expression of these cyclases has been demonstrated using insect *Spodoptera frugiperda* (Sf9) cell culture [50], yeast (*K. phaffii* [71], *S. cerevisiae* [37,40]), plant (*N. benthamiana* [38,50,74], and also claimed in a number of patents and patent applications. In all above systems, active enzymes can be produced without complications. However, the in vivo activity could be significantly improved by a number of bioengineering approaches as seen in two recent reports. Zirpel et al. [72] systematically examined the cyclase activity in yeast *K. phaffii* crude lysate by site-directed mutagenesis. Replacement of all seven Asn residues deemed necessary for *N*-glycosylation to Gln resulted in undetectable THCAS activity in cell lysate, whereas N89Q_N499Q double substitutions instead resulted in 2-fold increase of THCAS activity. At the enzyme active site, the triple substitution A414V_A46V_T47A was able to increase CBDAS activity 4.1 fold. The same group also looked into improving cyclase activity by enhancing protein folding in the ER [42]. ER chaperones (KAR2 and CNE), FAD synthetases (FAD1), foldases, protein disulfide isomerases (PDI1), and transcription factors (HAC1) were overexpressed in *K. phaffii*, which led to an impressive 20-fold in vivo THCAS activity increase. The result joins other reports in suggesting that improving ER protein folding is a viable solution to higher protein titer. It should be noted that the THCAS signal peptide was replaced with a vacuole protease A signal peptide in the above work to direct THCAS inside of vacuole instead of being secreted.

A systematic method for cannabinoid production engineered in yeast *S. cerevisiae* was also demonstrated in a patent application with similar approaches [36]. CBDA biosynthesis with full-length CBDAS harboring its native signal peptide was enabled by feeding OA to the yeast strain which was engineered with GPP production and other necessary cannabinoid biosynthetic genes (Table 1). Overexpression of proteins enhancing ER protein folding dramatically increased the basal CBDA titer of 5 mg/L (3-day feeding) to 179 mg/L (7-day feeding) with overexpression of KAR2, IRE1 (HAC1 activation), PDI1, ERO1 (PDI1 activation) and downregulation of ROT2 (ER glucosidase), PEP4 (protease). Site-directed mutagenesis in CBDAS also positively influenced enzyme activities shown by the improvement from the basal 162 mg/L to 234 mg/L (N196Q), 229 mg/L (L132M), or 224 mg/L (N57D) collected from testing 68 variants. Approximately 6–10% of the CBDAS product is THCA in these experiments. Similarly, THCA titer from OA feeding was improved from 131 mg/L with wild-type THCAS to 320 mg/L with N196Q substitution consistent with previous finding (Table 1) [72]. It is worth noting that wild type and most tested THCAS variants expressed in *S. cerevisiae* did not produce detectable amounts of CBCA, which was quite different from the THCAS produced from *K. phaffii* (10% CBCA) [72].

## 3. Precursor Geranyl Pyrophosphate Production

Prenylation of OA requires the supply of the C10 isoprenoid GPP. Isoprenoids, or terpenoids, are the largest class of natural products involved in both the primary and specialized metabolism in all three kingdoms of life, and all of them derive from simple C5 building unit isopentenyl pyrophosphate (IPP) and its *cis*-isomer dimethylallyl pyrophosphate (DMAPP) [82].Based on the number of isoprene units in the structure, primary isoprenoids can be classified into monoterpene (C10), sesquiterpene (C15), diterpene (C20), triterpene (C30), and other higher terpenes with more isoprene units, such as natural rubber which comprises tens of thousands of isoprene units. Isoprenoids are often cyclized by terpene synthases and further decorated to have impressive complexity, which is the driving force for their diversity and exciting bioactivities in nature. Simple isoprenoids also participate in central cell metabolism such as tRNA genesis (uracil modification), mitochondrial electron transport (*via* ubiquinone synthesis), and protein glycosylation and cell wall biosynthesis (*via* dolicol or undecarprenol synthesis). As a result their dynamics in cells are tightly regulated.

IPP and DMAPP are produced either from the mevalonate (MVA) pathway or the 2-*C*-methyl-D-erythritol 4-phosphate (MEP) pathway, named after respective signature intermediates (Figure 2). The MVA pathway is mostly found in the cytosol of eukaryotes, whereas the MEP pathway is found in most prokaryotes and the plastids of plants. In plants, GPP is synthesized primarily via the plastidial MEP pathway since the GPPSs are localized in plastids. In contrast, the biosynthesis of farnesyl pyrophosphate (FPP) that gives rise to all sesqui- and triterpenoids (e.g., sterols) in plants is primarily localized in the cytosol where the FPP synthases (FPPS) are found. For the popular heterologous production systems; the prokaryote *E. coli* contains the MEP but not the MVA pathway, whereas the eukaryote yeasts have the MVA but not the MEP pathway.

In the past two decades, extensive studies have been put into improving the isoprenoid production in microorganisms for their commercial uses in pharmaceutical and chemical industries. While several key points are explained below, more details are found in many excellent reviews [82,83,84,85]. In principle, engineering the isoprenoid production includes overexpressing the respective pathway enzymes, reducing the product feedback inhibition by enzyme engineering or bypassing these steps with alternative enzymes, reducing the consumption of the intermediates from competing metabolism by gene deletions, and other considerations such as stoichiometry, cell growth and process engineering. HMG CoA synthase (HMGS) and HMG CoA reductase (HMGR) are well-known rate-limiting enzymes in the MVA pathway, which are regulated both transcriptionally and post-translationally, whereas the gateway enzyme 1-deoxy-D-xylulose-5-phosphate synthase (DXPS) in the MEP pathway is inhibited by IPP. Partial or full MVA pathways have been frequently expressed in *E. coli* to circumvent MEP pathway regulation. In yeast, very commonly the HMGS is expressed as a *N*-terminal truncated version to remove the inhibitory domain. Overexpression of the gateway enzyme acetoacetyl CoA thiolase (ACCT) is also used to boost flux into the formation of acetoacetyl CoA in the MVA pathway (Figure 2). Many successes have been made in production of a number of isoprenoid compounds such as the sesquiterpene artemisinic acid in yeast (25g/L) and its precursor amorpha 1,4-diene in *E. coli* (27 g/L) with fed-batch bioreactor reaching industrial levels [86,87].

It should be noted that monoterpene production such as GPP in yeast remains challenging compared with the high titers of sesquiterpenes that are achievable in the same system [88]. *S. cerevisiae* barely produces monoterpenes nor does it contain a GPPS [89]. Its FPPS ERG20 and its products (ergosterols, dolichols etc.) are essential for yeast survival. ERG20 catalyzes consecutive additions of IPP onto DMAPP to form GPP then FPP, while the GPP intermediate remains enzyme bound. The effect of GPPS overexpression in yeast is hindered by ERG20 that effectively converts GPP further to FPP [90]. The double mutant *erg20* F96W_N127W and single mutant K197E have been developed to augment ERG20′s GPPS activity while reducing its FPPS activity, which led to several magnitudes of monoterpene titer improvement to mg per litre levels [90,91]. This is pertinent to GPP supply in yeast cannabinoid production, since it was reported that overexpressing mutant *erg20* outperformed overexpressing plant GPPS [35].

In the several related reports for cannabinoid production in yeast [35,36,37], the GPP production has been heavily engineered with one of the variations shown in Figure 2. In general, all MVA pathway genes were overexpressed with strong inducible promoters, and several could be replaced by enzymes from other organisms for higher activity or less inhibition in yeast. With CsPT4 and enhanced expression of terminal cyclases, these yeast strains were shown produce 200–300 mg/L CBGA, THCA or CBDA in shake flasks after 3–5 days when fed with exogenous OA (Table 1). Roughly 70–90 mg/L C10 precursor was produced based on stoichiometry. While it is difficult to unambiguously identify the rate limiting step based on the available reports, it is likely that GPP supply could still be a limiting factor in these strains considering the reported monoterpene titers in literature are in similar range [84].

An alternative solution has been devised to circumvent ERG20′s intrinsic GPP isopentanyltransferase activity, by introducing a neryl diphosphate synthase (NPPS) [88]. NPP is the *cis*-isomer of GPP and no longer a substrate for ERG20. Protein engineering on a terpene synthase permitted its use of NPP as substrate [88], which provides an interesting route for monoterpenoid production in yeast. CsPT1 does not accept NPP substrate [60], whereas the NPP preference of CsPT4 or StNphB has not been reported. The cyclases, however, are able to produce respective final cannabinoids from nerylated OA: CBNRA. As such, it may be possible to produce cannabinoids via NPP, if the prenyltransferases are found or engineered to use NPP or alternative prenyltransferases could be sourced.

Another alternative route for IPP or GPP supply is to provide exogenous isoprenol and prenol then convert them to IPP and DMAPP by overexpression of several kinases [92]. While it circumvents the endogenous regulation and perhaps limitation of isoprenoid precursor supply, its application in microbial cannabinoid production may be limited by other factors including GPP production, downstream enzyme activities, and feedstock cost.

There are few publicly available reports on engineering *E. coli* for cannabinoid production, although *de novo* synthesis of CBG or CBGA is possible since all enzymes to this point are either native to *E. coli* or can be effective expressed. For example, the monoterpene limonene could be produced in *E. coli* at 400 mg/L in shake-flasks enabled by the overexpression of MVA pathway and truncated plant GPPS [93]. A patent application describes overexpression of several MEP pathway genes including a bifunctional IspDF gene in *E. coli* towards IPP production, however the production of cannabinoids was not reported despite demonstrating functional expression of CsPT1 [43]. In another patent, detectable CBGA was claimed *de novo* in *E. coli* with cannabis enzymes and CsPT1 [46]. There are a number of companies claiming technology for cannabinoid production in *E. coli*, which may suggest adoption of the described approaches or inventions that are not made public.

## 4. Precursor Hexanoyl CoA Production

The origin of hexanoyl CoA in *C. sativa* has not been clearly determined. It is possible that hexanoate is produced via an early termination of chain-elongation during long-chain fatty acid biosynthesis. However, transcriptomic data supports the biogenesis of hexanoate through the fatty acid degradation pathway, in which a long-chain fatty acid is desaturated by a desaturase, oxidized by a lipoxygenase, and finally cleaved by a hydroperoxide lyase to give the free hexanoate (Figure 2). Members of these three enzymes were found to be highly represented in trichome transcriptomes [8,94]. Hexanoate subsequently forms the CoA thioester by one or more CoA ligases termed acyl-activating enzyme (AAE) or hexanoyl CoA synthase (HCS) found in *C. sativa*, such as CsAAE1-3 (CsHCS1-3) (Figure 2) [94,95]. CsAAE1 is likely responsible for hexanoic CoA biosynthesis *in planta* owing to its high transcripts and preference for medium-chain fatty acids [94]. While CsAAE1 and CsAAE2 appear to reside in the cytoplasm, CsAAE3 contains a peroxisome targeting sequence, which may suggest other *in planta* function [94,95].

Engineering the production of medium-chain fatty acids and alcohols in microorganisms predated the engineering of cannabinoid production, due to their implication as biofuels to replace fossil fuels. For example, the bacteria *Clostridium acetobutylicum* naturally produce 1-butanol (C4). The fermentative production of butanol is well understood in these bacteria and has been engineered either in *Clostridium* or *E. coli* using their enzymes, reaching titers of 10–30 g/L [96,97]. The pathway proceeds via acetyl CoA acetylation, ketone reduction, dehydration, and reduction reactions to form butyryl CoA, followed by thioesterification and carboxylate reduction to butanol [97]. Inclusion of a *Ralstonia eutropha* β-ketothiolase (RsBktB) allowed further extension of the C4 CoA ester to afford hexanoyl CoA (C6) from the same set of enzymes (Figure 2) [98]. Engineered *E. coli* afforded ~400 mg/L hexanol [99], while engineered yeast *Kluyveromyces marxianus* in a similar fashion was able to produce 140 mg/L hexanoic acid [44]. The same pathway was also used in a patent for cannabinoid production [51].

Compared to engineering GPP supply, engineering *de novo* hexanoyl CoA supply has not been actively explored for cannabinoid production in microorganisms. Several reasons may have contributed to the engineering decisions. Firstly, the production of hexanoyl CoA and IPP may compete for the same acetyl CoA pool if the MVA pathway is used. Secondly, exogenous hexanoate supply are sufficient for hexanoyl CoA biosynthesis with CsAAE overexpression seen in several aforementioned reports. Considering the lower price of hexanoate compared to IPP, it is more economically viable to use hexanoate as feedstock. Thirdly, the cannabinoid pathway enzymes (AAE, TKS, OAC, prenyltransferase and cyclases) are promiscuous enough to allow various short to medium aliphatic acids as starter molecules, rendering a repertoire of diverse cannabinoid analogs to be synthesized. Therefore, aliphatic acids feeding may be a wise choice for producing specialty cannabinoids.

## 5. Choice of Chassis and Further Technical Development

Various organisms have been attempted for the heterologous production of cannabinoids or precursors, including bacteria *E. coli* [43,45,46,47,65,66] and *Zymomonas mobilis* [100], cyanobacteria *Synechocystis* spp. [101] and *Synechococcus elongatus* [53], fungal species *S. cerevisiae* [33,34,35,36,37,40,41,51], *Kluyveromyces marxianus* [51], *Komagataella phaffii* (formerly *Pichia pastoris*) [40,41,42,72] and *Candida viswanathii* [52], and plant species *Chlamydomonas reinhardtii* (green algae) [53] and *Nicotiana benthamiana* (tobacco) [38,48,49,74] (Table 1). For metabolic engineering, *E. coli*, *S. cerevisiae,* and tobacco are among the most studied species. There are abundant molecular and genetic tools for their manipulation and decades of knowledge on their uses in biotechnology.

In these three species, *E. coli* is disadvantaged because the terminal cannabinoid cyclases could not be successfully expressed due to the requirement of eukaryotic post-translational modification system for proper protein folding. However, the prenyl and aliphatic precursors could be produced at gram per liter levels in *E. coli*, and CBGA production was possible with CsPT1 and StNphB. The future of using *E. coli* to produce cannabinoids relies on whether medical or nutraceutical uses of the non-cyclized cannabinoids will be developed, or if novel *E. coli* compatible enzymes that are able to cyclize cannabinoids are discovered. Alternatively, *E. coli* may be co-cultured with other organisms such as yeast to carry out the complete biosynthesis. The modular co-culturing strategy may overcome the limitations of the respective organisms, which has been demonstrated for the biosynthesis of several natural products [102]. However, significant engineering is needed for shuttling the intermediates between organisms and studying the co-culturing conditions to achieve high titers for industry use.

The yeast *S. cerevisiae* is well positioned for cannabinoid production. Extensive engineering of *S. cerevisiae* has allowed for 200–300 mg/L cannabinoid production from galactose and OA feeding, and 30–80 mg/L cannabinoid production if OA is replaced with hexanoic acid (Table 1). The lowered production suggest improvement in intracellular hexanoic acid availability as well as the improvement of CsTKS/OAS activity. The production of olivetol and methyl olivetol by a type I polyketide synthase provided an interesting route for cannabinoid biosynthesis and will certainly be viable to produce non-cyclized cannabinoids. However, the wild-type terminal cyclases do not accept CBG as a substrate, therefore this route is limited by the availability of a novel or engineered cyclase for converting the decarboxylated precursors. In addition, it appeared that cannabinoids were secreted to the growth media by the engineered yeast, since the analyses in the above reports were performed using whole culture extraction without mechanical cell lysis, however this requires further confirmation.

Plants are able to photosynthesize and therefor do not require an exogenous carbohydrate feedstock. They contain the necessary cellular machinery for expressing plant proteins that may need membranous systems or post-translational modifications that may not be provided by *E. coli* or yeast. Plants also contain plastids and vacuoles that may be explored for specialized metabolite production and accumulation. On the other hand, plant metabolism is more complex, which may require more engineering efforts for metabolite stability and storage to avoid toxicity and to improve yield. When CsPKS, CsOAC, and CsAAE1 were transiently expressed in tobacco, feeding hexanoic acid led to the formation of OA-4-*O*-glucoside as a major product. Unfortunately, further inclusion of CsPT4 and THCAS did not lead to detectable amounts of CBGA or THCA, despite all enzymes proving functional in tobacco [38]. This results suggests that metabolic engineering of GPP supply, prenyltransferase activity and subcellular localization, and the subcellular localization of THCAS (apoplast) are required to increase the final yield. The results are also consistent with other reports on the glycosylation of various cannabinoids in tobacco cells [53,101], and the requirement of ER or apoplast targeting sequence for its proper expression and function [74].

Plants are well known for their production of various glycosides of specialized metabolites as means to reduced toxicity and reactivity. Glycosylation significantly increases the water solubility of the otherwise hydrophobic cannabinoids and may allow higher accumulation of the glycosides in host plants. Similarly, cannabinoid glycosides will have higher solubility in human plasma, and may offer different bioactivities with the structural modification. However, the human metabolism of these glycosides is unknown, and their reduced lipophilicity may reduce their diffusion across the blood brain barrier if central nervous system targeting is desired.

In addition to the in vivo production systems, in vitro cell free systems have also been engineered for cannabinoid biosynthesis. A heavily engineered system was used to produce GPP, which was used subsequently to prenylate OA feedstock. With soluble StNphB engineered for higher specificity and turnover rate, the system was able to produce 1.25 g/L CBGA after 100 h [55,56]. The system was further simplified by using alternative enzymes for GPP production and was combined with enzymes responsible for hexanoyl CoA biosynthesis. The system produced less CBGA (480 mg/L), but was able to achieve it in less time (10 h) and with less expensive feedstock (hexanoic acid) (Table 1) [54,55].

Almost all current recombinant cannabinoid producing systems use the cannabis genes including CsAAE1-3, CsTKS, CsOAC, CsPT1/4, and THCAS/CBDAS/CBCAS. Engineered NphB provides an alternative to circumvent the dependence of membrane-bound cannabis prenyltransferases. Other membrane-bound aromatic prenyltransferases are also found to prenylate the resorcinyl core, including StbC from the fungus *Stachybotrys bisbyi* and *Rhododendron* RdPT1 that farnesylates osellinic acid [15,103], which may be engineered to perform geranylation. Olivetol biosynthesis can be achieved by other PKSs; however, new enzymes are needed to cyclize oliveto-based cannabinoids since the activity of the original cyclases depends on the carboxylate group. Further discovery and enzyme engineering may provide alternative methods for producing these cannabinoids.

## 6. Future Remarks

The acceptance of cannabis and cannabinoid products has been evolving with the knowledge gained from the studies of the human endocannabinoid system, however it is highly complexed with the societal experiences from decades of use and abuse. The CB1 receptor modulates retrograde neuron inhibition and its homeostasis is disturbed in several neurological disorders such as Parkinson’s Disease and Alzheimer’s Disease. In addition to their interaction with CB1, THC, THCV, and CBD have been found to have modulatory effect on other receptors in neurons and immune cells, such as the nociceptive receptor TPRV1. These discoveries support developing medicines and treatments based on the existing cannabinoids and the cannabinoid pharmacophore. Sativex^®^ (Nabiximols, THC:CBD = 1:1) has been approved for treating spasticity caused by multiple sclerosis in the UK, Canada, and Australia. Epidyolex^®^ (pure CBD) has been approved in the US and EU for treating several rare forms of seizures (Lennox-Gastaut syndrome, Dravet syndrome, and tuberous sclerosis complex). These approvals are based on reported clinical efficacy. On the other hand, the increasing amount of knowledge gained in the endocannabinoid system and its regulation also suggests that various receptors and signaling pathways may be used for disease intervention.

Does this mean that the cannabis-derived phytocannabinoids are the solutions to the many diseases where they may have an implication? To answer this complex question, pharmaceutical companies must demonstrate the effectiveness and safety of phytocannabinoid based drugs through clinical trials, where many experimental drugs fail to address either or both despite the effectiveness in cellular and animal models. The legalization of both recreational and medical uses of cannabis in Canada and several US states, and the anticipation of the legalization at the US federal level has been propelling the rapid development of the legal cannabis industry, which is looking to maximize the value of cannabis. As such, there is a strong demand to develop cannabis and cannabinoids into medicines and nutraceuticals due to potential financial benefit. However, the psychotropic effect of THC is a major concern and drawback for its medicinal development. Other non-psychotropic cannabinoids such as CBD, or even other cannabis phytochemicals such as various terpenoids, may be viewed as safe compounds for further pharmaceutical development. With both the push from the industry and pull from the medical research, we may see positive results in the medical field in the years to come.

Cannabis is a remarkable plant capable of producing impressive amounts of cannabinoids exceeding 20% of the flower bud dry weight, which makes it one of the most productive plants for accumulating specialized metabolites. In comparison, the antimalarial drug artemisinin and precursors are produced at less than 3% leaf dry weight from the plant *Artemisia annua* [104], much lower than the yield of cannabis. Despite the invention of a synthetic biology marvel capable of producing 25 g/L artemisinic acid in yeast, the majority of world artemisinin is still harvested from plants due to the low price of plant artemisinin as a result of plant breeding, agronomy development, and the supply-demand self-regulation [105]. It will be a great challenge to rival the plant efficiency with microbial fermentation for producing the major cannabinoids such as THC and CBD, especially when significant resources have been put into cannabis agronomy studies and the whole sale price for these cannabinoids are steadily dropping since 2019 (https://hempsupporter.com/assets/uploads/USHRHempPricingData.pdf (accessed on 29 January 2021)).

However, the promise of heterologous cannabinoid production is its ability of producing rare and new cannabinoids for medical use, which the plant cannot easily afford without genetic modifications. The promiscuity of the cannabinoid pathway enzymes allows the substitution of the pentyl side-chain on the major cannabinoids with various aliphatic side-chains that impact the binding through altered van der Waals interaction. While the pyran ring, the free hydroxyl group, and the C11 methyl group are all important for THC-CB1 binding [106,107], modifications such as glycosylation and hydroxylation may further diversify cannabinoids and alter their agonism/antagonism. Synthetic biology therefore provides the ideal solution to such development by sourcing new enzyme catalysts, while metabolic engineering and fermentation technology will help the production achieve industrial level.

On the plant side, enzymes may still be discovered in cannabis for the biosynthesis of some rare phytocannabinoids (Figure 1), and their metabolic regulation is worth studying too. Further plant breeding may also allow improved production of these rare cannabinoids. However, the most important development in cannabis is perhaps to improve plant performance in the areas of disease resistance and agronomy that will increase overall cannabinoid production since the individual plants are likely maxed out metabolically in their cannabinoid formation capacity.

In the jurisdictions where recreational cannabis is legal, plant-derived cannabinoids will remain significant for the recreational use. Ultimately, the fate and sustainability of non-cannabis cannabinoids will depend on the medical development of the rare and new cannabinoids, the competition from synthetic and semi-synthetic cannabinoids, and perhaps most importantly, the demand from the market and companies’ business development.

## Figures and Tables

**Table 1:** Summarized published work of heterologous systems for cannabinoid production.

Expression Systems	Feedstock	Product	Titer	Sources
*Saccharomyces cerevisiae*	Olivetolic acid	CBGA	96 mg/L	[34]
	Olivetolic acid	THCA	84 mg/L	[34]
	Hexanoic acid	CBGA	34 mg/L	[34]
	Hexanoic acid	THCA	23 mg/L	[34]
	Olivetolic acid	CBGA	216 mg/L	[35]
	Hexanoic acid	CBGA	73 mg/L	[35]
	Olivetolic acid	CBDA	234 mg/L	[36]
	Olivetolic acid	THCA	320 mg/L	[36]
	*de novo*	1-methyl-CBG	66 mg/L	[30]
	*de novo*	Olivetol	76 mg/L	[30]
	*de novo*	1-methylolivetol	42 mg/L	[30]
	Hexanoic acid	CBDA	4.2 µg/L	[37]
	Hexanoic acid	THCA	8.0 mg/L	[37]
	Olivetolic acid	CBGA	1.0 mg/L	[38]
	Olivetolic acid	CBGA	1.0 mg/L	[38]
*Komagataella phaffi (previously Pichia pastoris)*	CBGA	THCA	32.6 mg/L	[39]
	CBGA	THCA	0.36 g/L	[40]
	Olivetolic acid	THCA	615 pmol/L	[41]
	CBGA	THCA	3.05 g/L	[42]
*Escherichia coli*	Olivetolic acid/GPP	CBGA	1.2 mg/L	[43]
	*de novo*	Hexanoic acid	140 mg/L	[44]
	*de novo*	Hexanoic acid	190 mg/L	[45]
	*de novo*	CBGA	detectable	[46]
	Hexanoic acid	Olivetolic acid	0.48 mg/L	[47]
*Nicotiana benthamiana*	CBGA	CBDA	34ppm	[48]
	CBDA	CBDA-glucoside	N/A	[48]
	CBDA	CBDA-glucoside	N/A	[49]
	CBGA	THCA	N/A	[38]
	Hexanoic acid	Olivetolic acid glucoside	N/A	[38]
	Hexanoic acid	Olivetolic acid	N/A	[38]
	CBGA	THCA	82 µg/30 mL	[50]
*Kluyveromyces marxianus*	*de novo*	CBDA, THCA	N/A	[51]
*Candida viswanathii*	oleic acid	CBGA	0.67 mg/L supernatant 1.51 mg/L lysate	[52]
	oleic acid	Olivetolic acid	13.1 mg/L	[52]
*Chlamydomonas reinhardtii*	*de novo*	CBGA	detectable	[53]
Cell free	Olivetolic acid	CBGA	744 mg/L	[54,55]
	Olivetolic acid	CBGA	1.25 g/L	[54,56]
